# Histone deacetylase 6 (HDAC6) is an essential factor for oocyte maturation and asymmetric division in mice

**DOI:** 10.1038/s41598-017-08650-2

**Published:** 2017-08-15

**Authors:** Dongjie Zhou, Yun-Jung Choi, Jin-Hoi Kim

**Affiliations:** Department of Stem Cell and Regenerative Biotechnology, Humanized Pig Research Center (SRC), Konkuk University, Seoul, 143-701 Republic of Korea

## Abstract

Tubastatin A (Tub-A), a highly selective histone deacetylase 6 (HDAC6) inhibitor, has been widely used as a cytotoxic anticancer agent, or for the treatment of patients with asthma. However, the potential toxicity of Tub-A on oocyte maturation and asymmetric division is still unclear. Therefore, the present study was designed to examine the effect and potential regulatory role of Tub-A on the meiotic maturation of oocytes. We observed that Tub-A treatment induced an increased level of the acetylation of α-tubulin, and a failure of spindle migration and actin cap formation. Based on the spindle structure, most Tub-A treated oocytes were arrested in an MI-like or a GVBD-like stage and exhibited decondensed chromosomes in a dose dependent manner. Moreover, Tub-A treatment decreased the protein expression of mTOR, a factor responsible for spindle formation, and the expression of mDia1, an inhibitor of actin assembly, in an HDAC6 expression-dependent manner. Importantly, following Tub-A supplementation, most oocytes failed to extrude the first polar body, which indicates that these defects are closely linked to abnormal oocyte maturation. Taken together, our data demonstrates that HDAC6 is one of the essential factors for oocyte maturation and asymmetric division via the HDAC6/mTOR or mDia1 pathway in mice.

## Introduction

In mammals, oocyte maturation requires a precise and orderly multistage process^1, 2^. During the process of oocyte maturation, the oocytes initiate spindle organisation and positioning, and the establishment of cortical polarity, which are essential steps preceding asymmetric division^3^. After germinal vesicle breakdown (GVBD), microtubules organise into a specialised barrel-shaped bipolar spindle, with all the chromosomes aligned at the spindle equator^4^. The spindle moves to the subcortical area that forms a thickened F-actin cap surrounded by a myosin II ring^5^. Moreover, the decrease in cortical tension required for spindle positioning is fine-tuned by a branched F-actin network which triggers the delocalisation of myosin-II from the cortex^6^. Thereafter, oocytes extrude the first polar body and arrest at the metaphase II stage until fertilisation occurs^7^.

Mammalian histone deacetylases (HDACs) are divided into four classes: class I (HDACs 1, 2, 3, and 8), class II (HDACs 4, 5, 6, 7, 9, and 10), class III (SIRTs 1, 2, 3, 4, 5, 6, and 7), and class IV (HDAC11)^8, 9^. Currently, it is well-known that histone deacetylase 6 (HDAC6) is a unique member of class II b HDACs, with two catalytic domains and a predominantly cytoplasmic localisation^10^. This HDAC isoform regulates various cellular processes, including microtubule-based transport, cell motility^10, 11^, endocytosis^12^, cell migration^13^, autophagy^14^, aggresome formation^15, 16^, neurotransmitter release^17^, vesicle^18^, mitochondrial transport^19^, glucocorticoid receptor maturation^20^, protein turnover^21, 22^, and degradation^23, 24^ by deacetylating non-histone proteins, such as α-tubulin and cortactin^25^. Increasing evidence demonstrates that HDAC6 biallelic knock-out (bKO) male mice can survive to adulthood, indicating that tubulin hyperacetylation is not a critical factor for male mammalian development^26^. Male HDAC6 bKO mice show hyperactivity in the open field test, less anxiety in the elevated plus-maze test, antidepressant-like behaviours in the tail suspension, and forced swim tests^27^. Of note, the deletion of the *HDAC6* gene rescues ciliary defects induced by Cyld loss in the testis, trachea, and kidney, without affecting other organs^28^. To date it has not been established whether second generation of homozygous female HDAC6 bKO mice successfully undergo oocyte meiosis and asymmetric division. This is very important issue for the successful generation of homozygous offspring.

Tubastatin A (Tub-A) is a potent and highly selective HDAC6 inhibitor^29^. Daily intraperitoneal (i.p.) injection of 25 mg/kg Tub-A into mice for 20 days neither affected brain morphology, brain/body weight mass, liver enzyme measurements, nor kidney function^30^. *In vitro*, even though Tub-A has no significant side effects on normal cells, a previous study demonstrated that the selective inhibition of HDAC6 can promote hyperacetylation of α-tubulin and decrease cell motility^10, 11^. This area of study is important because histone deacetylase inhibitors can be used as a treatment for airway remodelling in patients with asthma^10^. However, little is known regarding the potential toxicity of Tub-A on oocyte maturation and asymmetric division in the context of animal studies. Therefore, this study was aimed to investigate the influence of HDAC6 on oocyte meiotic maturation and asymmetric division following treatment with Tub-A.

## Results

### Tubastatin A blocks polar body extrusion in mouse oocytes

**Figure 1:**
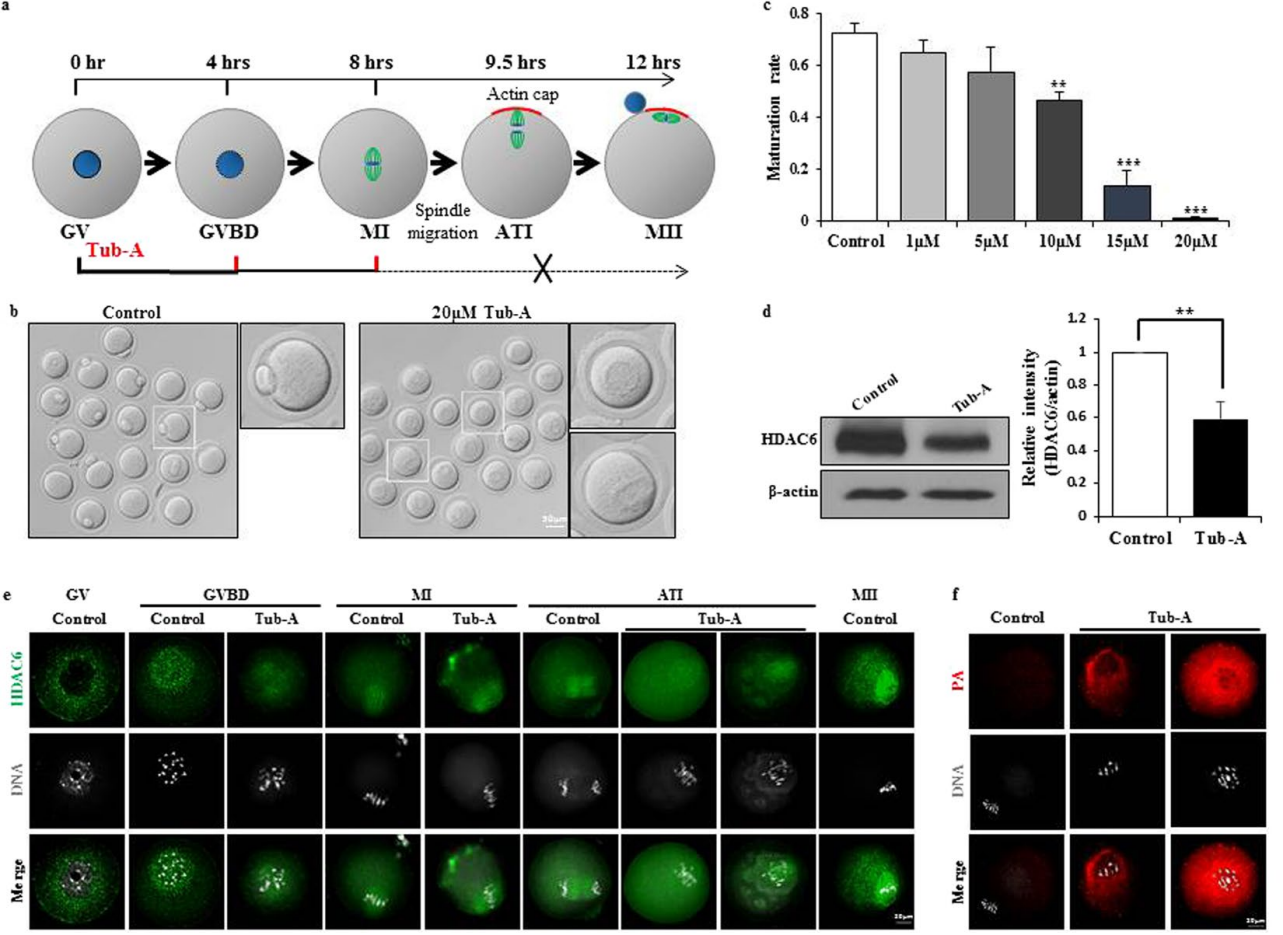
Effect of Tub-A on oocyte maturation. (**a**) Tubastatin A treatment time course during oocyte maturation. Blue circle, nuclear; green line, spindle; red line, actin cap. (**b**) Images of MII stage oocytes in the control and Tub-A treatment group. (**c**) Quantitative analysis of the first polar body extrusion rate after 12 h of in vitro culture. Graph shows means ± SD of results obtained in three independent experiments. (**d**) Western blot showing partial knockdown of HDAC6 after treatment with 20 μM Tub-A. (**e**) Confocal imaging analysis of HDAC6 localisation during oocyte meiosis. Oocytes at GV, GVBD, MI, ATI, MII stages are immunolabelled with HDAC6 antibody (green) and counterstained with TO-PRO-3 to visualise DNA (gray). (**f**) Confocal images of aggresome formation levels in oocytes matured in the presence of Tub-A. Red, protein aggregates; Grey, nuclear. The rate is significantly different (**p < 0.01; ***p < 0.001).

We first examined the effects of tubastatin A (Tub-A) on mouse oocyte maturation and asymmetric division, as visualised by light and confocal microscopy. To examine the functional roles of HDAC6 during oocyte meiotic maturation, we treated germinal vesicle (GV) oocytes with five different Tub-A concentrations for 12 h (Fig. 1a). Most control oocytes extruded the polar body and developed to the MII stage (Fig. 1b, left), whereas, as shown in the right panel of Fig. 1b and in Fig. 1c, treatment with 20 μM Tub-A resulted in the failure of polar body extrusion. As shown in Fig. 1c and Supplementary Figure 1a, the ratio of polar body extrusion in control oocytes was 72.57 ± 3.74% (*n* = 476). When oocytes were treated with Tub-A at a concentration of 1 µM, 5 µM, 10 µM, 15 µM, and 20 µM, the ratio dramatically decreased from 65.03 ± 4.86% (*n* = 165; *p* = *0.011193*) to 57.28 ± 10.02% (*n* = 177; *p* = 0.083494), 46.37 ± 3.41% (*n* = 143; *p* = 0.001941), 13.57 ± 5.95% (*n* = 162; *p* = 0.004428), and 1.17 ± 0.76% (*n* = 447; *p* = 0.000547), respectively. Therefore, our observations suggest that Tub-A treatment reduces polar body extrusion in a dose-dependent manner.

To identify the underlying mechanism of Tub-A function, 20 μM Tub-A-treated oocytes were subjected to further study. Of note, Western blotting analysis using anti-HDAC6 antibody showed that Tub-A treated oocytes for 12 h significantly decreased the expression of HDAC6 protein (Fig. 1d), indicating that HDAC6 directly target HDAC6 gene itself to regulate its expression^31^. To further define the cellular events in which HDAC6 is involved during the meiotic maturation, we examined its localisation at different stages of the mouse oocyte maturation by indirect immunofluorescence microscopy (Fig. 1e). In control oocytes, HDAC6 expression in GV-stage oocytes uniformly resided in the cytoplasm of oocytes. At the GVBD stage, the HDAC6 staining patterns started to migrate in the nuclear area respect to the surrounding the chromosomes. As the oocytes entered metaphase, HDAC6 staining is gradually increased along with the spindle region. During the anaphase and telophase stages, intense fluorescence signals of HDAC6 are detected into a spindle like pattern and then move to the cortex region. However, localization of HDAC6 expression after Tub-A treatment was mainly limited to nucleus including weakly and sparsely spot of cytoplasm at GVBD stage and spindle at MI and ATI (Fig. 1e). Likewise, the Tub-A treated group showed very strong protein aggregation (PA) staining, indicating that Tub-A treatment may result in cell death due to the significant accumulation of PA (Fig. 1f).

### Tub-A causes the failure of cytokinesis in oocyte meiosis

**Figure 2:**
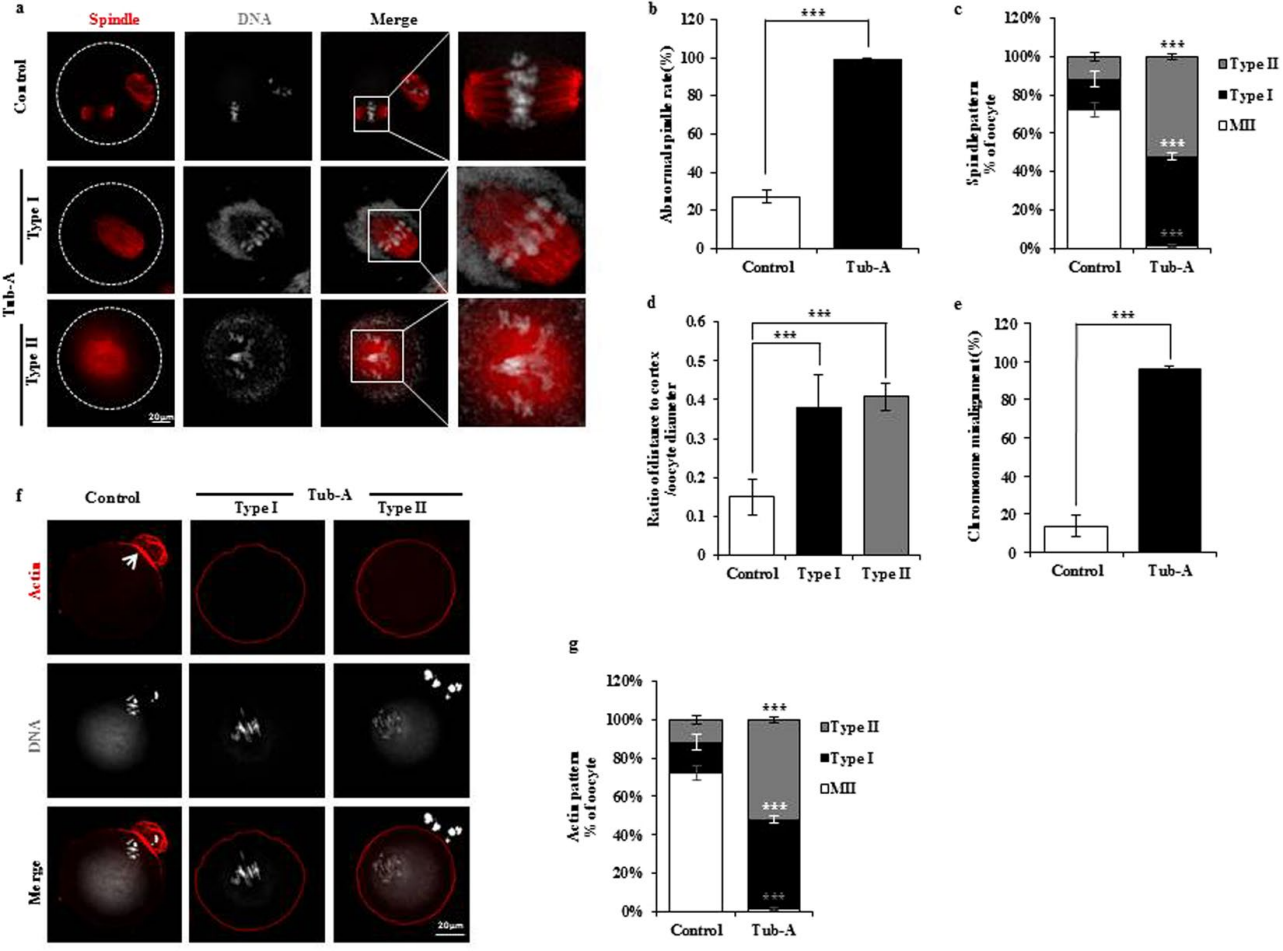
DMSO treatment disrupts spindle migration, positioning, and actin cap formation. (**a**) Oocytes were stained with α-tubulin antibody to visualise the spindle (green) and counterstained with TO-PRO-3 to visualise chromosomes (red). The white circle shows the oocyte boundary. Red bar, distance from the centroid of the spindle to the cortex; Blue bar, diameter of the oocyte. (**b**,**c**) Quantification of control and Tub-A-treated oocytes with spindle defects or percentage of Tub-A treated oocytes of different types. Data are expressed as mean ± SD percentage from three independent experiments in which >50 oocytes were analysed. (**d**) The distribution of spindle locations in Tub-A treated and control oocytes. Spindle location is expressed as red bar length/blue bar length, where red bar is the distance between the chromatin and the cortex, and the blue bar is the oocyte diameter, as indicated in (**a**) n values are as indicated. ***p < 0.001. (**e**) Tub-A treatment during meiotic maturation significantly increases the proportion of oocytes with aberrant chromosome morphology compared to controls (*p* = 0.000547). (**f**) Representative images showing the actin distribution in control and Tub-A treated oocytes. Arrowheads indicate the position of the actin cap. Red, actin; Grey, chromosome. (**g**) Quantification of control and Tub-A treated oocytes with different actin patterns in the mouse oocyte.

In mammalian oocytes, spindle migration is driven by actin^32–34^. Using confocal microscopy, we examined the spindle and actin morphologies in oocytes after 12 h of *in vitro* maturation. In the control group, a small polar body and a large MII oocyte had been formed. Most oocytes in metaphase II presented typical barrel-shaped spindles, which were located under the region of the cortex where the actin cap had been formed (Fig. 2a). In contrast, in the Tub-A treated group, spindle defects were readily observed at high frequency (Fig. 2b), and were characterised by MI-like stage (Type I) and GVBD-like stage spindles (Type II) (Fig. 2a,c). Compared with the control oocytes (15.7 ± 3.93% and 11.73 ± 1.99%, respectively), the rates of Type I and Type II in Tub-A treated oocytes were significantly increased (46.82 ± 1.79% and 52.02 ± 1.45%; *p* = 0.003181 and 0.001038, respectively). In most of oocytes, the spindle failed to migrate to the cortex after treatment with Tub-A (Fig. 2d). In contrast to control oocytes, in which condensed metaphase chromosomes aligned along the equatorial plane, exposure to Tub-A induced the formation of elongated chromosomes (Fig. 2a,e).

As shown in Fig. 2f, actin staining using phalloidin showed that control oocytes could form an actin cap. However, Tub-A treatment caused an apparent alteration in the arrangement of the actin cytoskeleton. Immunofluorescence analysis showed that the actin signals in the Tub-A treated oocytes displayed different patterns when compared with control oocytes (Fig. 2g). These results indicate that spindle migration and actin cap formation were disrupted after treatment with Tub-A.

### Tub-A increased the α-tubulin acetylation level in oocytes

**Figure 3:**
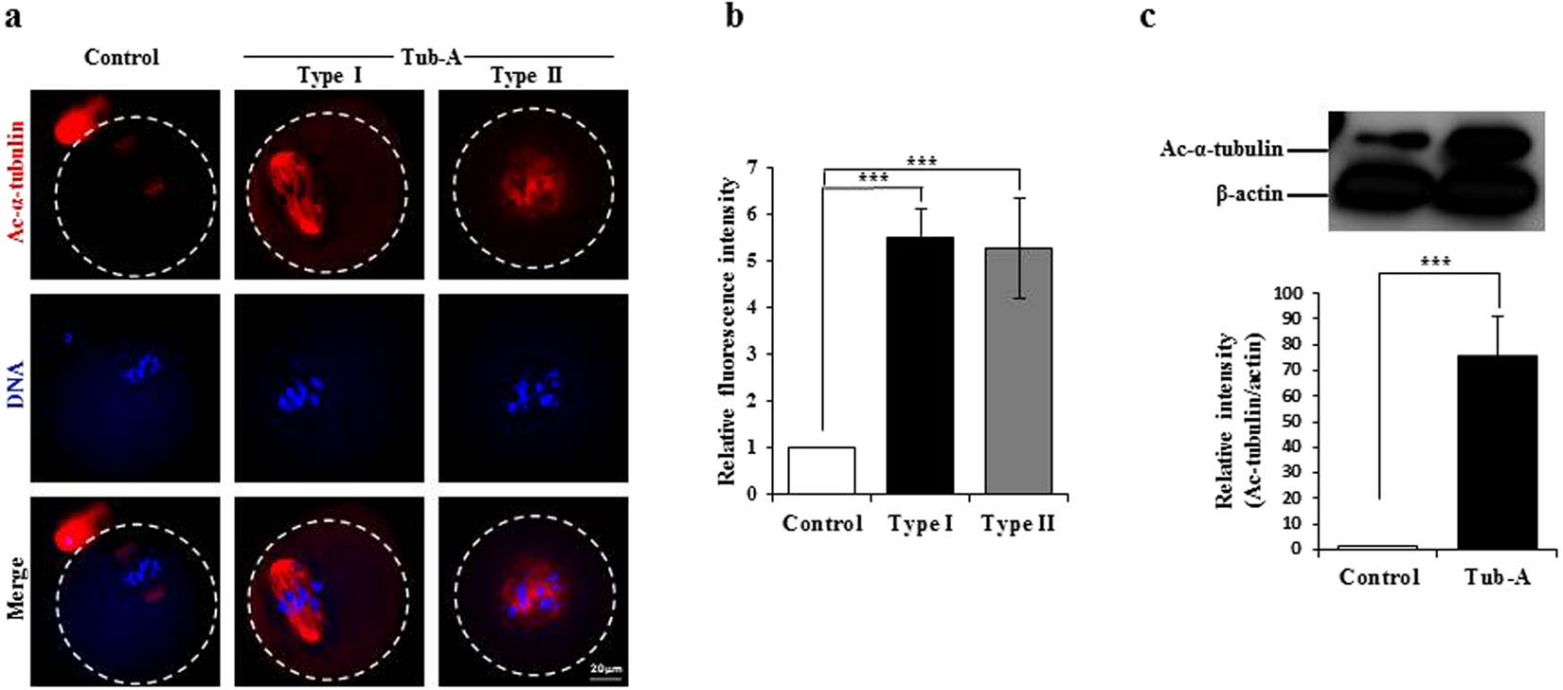
Increased acetylation of α-tubulin in Tub-A treated oocytes. (**a**) Representative images of acetylated α-tubulin in control and Tub-A treated oocytes. Red: acetylated α-tubulin; Grey: chromosome. (**b**) Quantification of the data shown in (**a**). Experiments were conducted three times, and over 50 oocytes were analysed for each group. (**c**) Western blot analysis showing the increased acetylation of α-tubulin in the Tub-A treatment group compared to the control group. Actin served as a loading control throughout. Band intensities were calculated using the ImageJ software; ratio of acetylated α-tubulin/actin expression was normalised, and values are indicated. ***p < 0.001.

It has been reported that Tub-A treatment leads to an increase in acetylated α-tubulin *in vitro* ^29^. α-tubulin acetylation serves as a marker for the presence of stable microtubules, and may affect the activity of microtubule-associated proteins and microtubule-based motors^35–38^. It is possible to speculate that HDAC6 regulates spindle function through the direct deacetylation of tubulin. To test this, we examined the effects of Tub-A on tubulin acetylation by staining oocytes with an antibody against acetylated-tubulin. As expected, we found that the acetylation levels of α-tubulin were significantly increased in the Tub-A treated oocytes compared with controls (*p* = 0.002) (Fig. 3). As shown in Fig. 3a, the abnormal bipolar spindle in Type I oocytes and the single round-shaped spindle in Type II oocytes showed very strong α-tubulin acetylation staining patterns, whereas the acetylated α-tubulin in control oocytes showed very weak staining around the bipolar spindle. These results indicate that the abnormal spindle morphology in Tub-A treated oocytes is closely associated with a high level of acetylated α-tubulin.

### HDAC6 functions in several pathways during mouse oocyte maturation

**Figure 4:**
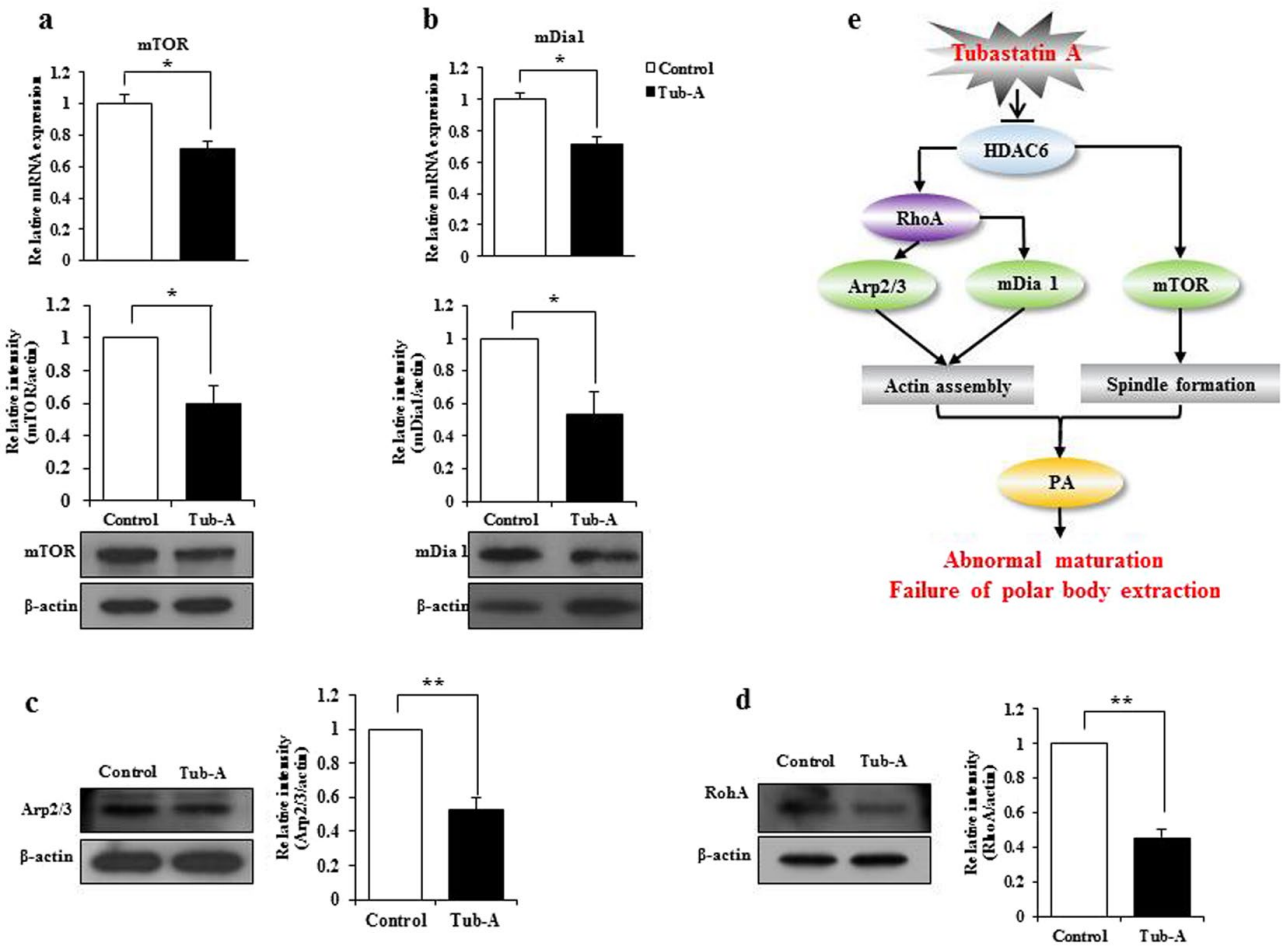
The effect of HDAC6 on several factors involved in meiosis. (**a**,**b**) RNA expression levels of mTOR and mDia1 in control and Tub-A treated oocytes after the 12 h treatment. Western blot analyses were performed to detect mTOR and mDia1. Values are indicated: *p < 0.05, **p < 0.01. (**c**,**d**) Arp2/3 and RhoA protein levels detected by western blot. (**e**) Proposed model to explain the role of HDAC6 during mouse oocyte meiosis.

To obtain further insights into the cellular pathways affected by the down-regulation of HDAC6 expression during the meiosis of mouse oocytes, we examined the expression levels of key regulatory factors involved in oocyte meiosis and asymmetric division. As shown in Fig. 1c and Supplementary Figure 1a, the effect of Tub-A on maturating oocytes is clearly dose-dependent. At 20 μM dose, oocyte maturation is completely arrested. RT-qPCR revealed that the mRNA expression levels for mTOR and mDia1 following treatment with Tub-A were significantly decreased. Western blot analysis also confirmed that the mTOR and mDia1 protein expression levels were significantly reduced after Tub-A treatment (Fig. 4a,b). Of note, key genes and proteins expression involved in actin assembly and spindle formation, such as Arp2/3 and RhoA, were significantly down-regulated following Tub-A treatment (Fig. 4c and d, respectively). These results may be caused by signaling pathway involved in downregulation of HDAC6 because HDAC6 could directly interact with HDAC6 genes and as a result, downregulate the expression of HDAC6 protein. However, the protein expression levels of PI3 kinase, p-AKT, and AKT were not substantially different between the Tub-A treated and the control group (Supplementary Fig. 1b,c). Also, the data showed that Tub-A treatment for 12 h did not block the extracellular signal, which regulates Kinase-1 and -2 (ERK1/2) phosphorylation and/or activation (Supplementary Fig. 1d). Therefore, we propose a key underlying mechanism for explaining the failure of meiosis in Tub-A treated oocytes (Fig. 4d). Taken together, our results suggest that HDAC6 might be essential for the regulation of actin assembly and spindle formation during oocyte meiosis via mTOR and mDia1 pathway.

## Discussion

This study demonstrated that the selective HDAC6 inhibitor, Tub-A, disrupted the spindle migration, actin cap formation, and asymmetric division during oocyte maturation. Moreover, we first demonstrated that HDAC6 expression is an essential factor for mouse oocyte maturation and provided direct evidence that HDAC6 is critically involved in the asymmetric division of the oocyte.

Previous study demonstrated that the endogenous HDAC6 expression in murine somatic cells such as FM3A, MEL, B16 cells, or Balb/c3T3 fibroblasts is mainly located in the cytoplasm^39^. However; its localisation has remained unclear during oocyte maturation. In the current study, our data showed that HDAC6 expression at the GV stage of control oocytes is mainly located in the cytoplasm. Our observation is line with the previous study found that HDAC6 protein expression is localised to the cytoplasm of the germinal vesicle^40^. At the GVBD stage, however, the HDAC6 protein formed aggregates and surrounded each chromosome. From the MI stage onwards, the HDAC6 protein expression was located to the spindle structure (Fig. 1e). Therefore, we hypothesised that HDAC6 is a critical factor for spindle formation during oocyte meiosis and the asymmetric oocyte division. To test our hypothesis, Tub-A, a potent and highly selective HDAC6 inhibitor^29^, were supplemented into culture medium for culturing of GV stage oocytes and oocyte maturation was counted at 12 h after Tub-A supplementation. As expected, the majority of Tub-A treated oocytes lost or showed a decrease in HDAC6 activity. Furthermore, these oocytes failed to extrude the oocyte polar body. Of note, even though we thoroughly washed Tub-A-treated oocytes with fresh medium to remove Tub-A, most oocytes pretreated with Tub-A for 12 h were not able to develop to the MII stage. Instead, the majority of oocytes pretreated with TubA were arrested at an MI-like or GVBD-like stage. Taken together, our observations strongly indicate that HDAC6 is a critical factor for spindle formation during oocyte meiosis and the asymmetric oocyte division.

To examine the quality of MI-like stage or GVBD-like stage oocytes, we examined the aggregates of ubiquitinated proteins (aggresomes) in these oocytes using a ProteoStat Aggresome Detection Kit. As shown in Fig. 1f, most Tub-A treated oocytes led to very strong aggresome staining patterns, whereas control oocytes showed very weak signals. Generally, protein aggregates do not accumulate in normal cells despite their continued production, because of the existence of a cellular ‘quality control’ machinery^15, 41^. As described in a previous review, the bulk of protein aggregates in intracellular and extracellular lesions are closely associated with cell death in many degenerative diseases^41, 42^. Lee *et al*. reported that HDAC6 promotes the fusion of autophagosomes and lysosomes^14^. In this study, Tub-A treated oocytes significantly decreased HDAC6 expression. Consistent with a previous finding^43^, Tub-A treated oocytes significantly reduced HDAC6 protein expression.

It is conceivable to speculate that control oocytes may remove the bulk of accumulated aggresomes through an autophagic route to protect the oocytes, whereas treatment with Tub-A triggers a significant accumulation of protein aggregates, indicating that HDAC6 inhibition in oocytes is irreversible. Taken together, we conclude that the accumulation of aggresomes in Tub-A treated oocytes might interfere with essential functions in oocyte meiosis, such as cytoskeletal organisation and the asymmetric oocyte division.

To determine the reason behind polar body extrusion defects following HDAC6 inhibition, we examined the actin filament distribution, which plays essential roles in oocyte polarity formation and cytokinesis^44^. Moreover, it is well known that the Arp2/3 complex^45^ and mDia1^46^ are involved in actin organisation during oocyte maturation in mice. The results of this study showed that the mDia1 mRNA and protein expression levels were significantly decreased, compared to those in the control (Fig. 4b). Further, following the inhibition of HDAC6 after Tub-A treatment, actin failed to form the cortical actin cap (Fig. 2d). Destaing *et al*.^47^ reported that Rho interferes with the osteoclast maturation process by controlling the level of microtubule acetylation and actin organisation through mDIA2 and HDAC6. Therefore, we tested and confirmed that RhoA was also inhibited by Tub-A (Fig. 4d). In conclusion, we suggest that mDia1 and RhoA dysregulation via the inhibition of HDAC6 may represent a possible pathway underlying the actin defects during oocyte maturation.

Previously, Lee *et al*.^48^ reported that the failure to form the actin cap disrupt spindle migration and lead to an abnormal asymmetric division during the meiotic maturation of oocytes, which was caused by a low expression of mTOR. In this study, we also detected a low level of mTOR expression in Tub-A treated oocytes. Previous study reported that HDAC6 protects neurons from toxicity of prion peptide, and that this protection occurs at through the regulation of the PI3k-Akt-mTOR axis^49^. However, the inhibition of HDAC6 during oocyte maturation did not alter PI3K and AKT protein expression (Supplementary Figure 1b and c). Of note, our results indicate that Tub-A treatment also decreased mTOR expression at both mRNA and protein levels by HDAC6 downregulation. The impact of HDAC6 on mTOR signaling could be linked to targeting of HDAC6 gene itself or its effect on α-tubulin acetylation. Taken together, these results suggest that dichotomous effects of HDAC6 on the HDAC6/mTOR or HDAC6/mDia1-mediated signaling pathways might inhibit spindle migration and asymmetric division of oocytes. Further, we observed a striking reduction in oocyte numbers with asymmetric division upon 12 h of HDAC6 inhibition that correlated well with the increase in α-tubulin acetylation. In conclusion, we believe that HDAC6 is an essential factor for cytokinesis and chromosome condensation in normal mouse oocytes. Thus, this study can provide important information for the development of safe and non-toxic HDAC6 inhibitors for animals and human beings.

## Methods

### Animals and reagents

The mice were housed in wire cages at 22 ± 1 °C under a 12 h light-dark cycle with 70% humidity and fed a regular diet. All experiments were conducted in accordance with the Konkuk University Guide for the Care and Use of Laboratory Animals (IACUC approval number: KU16122). Unless otherwise noted, all reagents for embryo culture were purchased from Sigma-Aldrich (St. Louis, MO, USA).

### Oocyte collection and culture

Hybrid B6D2F1 (C57BL/6 × DBA) female mice (6–8 weeks old) were sacrificed 48 h after the administration of pregnant mare serum gonadotrophin (PMSG) in 5 IU doses. GV oocytes were selected and maintained in M16 medium, and covered with sterile mineral oil under 5% CO2 at 37 °C.

### Tubastatin A treatment

Tubastatin A HCl (Catalogue no. 27108) was obtained from BPS Bioscience (San Diego, USA). The compound was dissolved in DMSO and further diluted in saline to the final concentration. Oocytes were collected and washed three times in M16 medium, and then randomly cultured in the same medium containing 1–20 μM Tub-A. Oocytes in the control group were incubated with the same amount of solvent alone (DMSO) for 12 h.

### Western blotting

A total of 15–50 oocytes were lysed in RIPA buffer (GenDOPET, Texas, USA) containing protease inhibitors and heated for 5 min at 100 °C. Total oocyte proteins were subjected to electrophoresis on a 10% SDS-PAGE gel. The separated proteins were transferred to PVDF membranes, which were pretreated with methanol. The membranes were blocked in 5% skim milk and incubated with primary antibodies as follows: HDAC6 (Santa Cruz Biotechnology Inc., Santa Cruz, CA, USA), Acetyl-α-tubulin (Thermo Fisher Scientifc, Rockford, IL, USA), mDia1 (BD Biosciences, San Jose, CA), and mTOR (2983), PI3 Kinase p110α (4249), Phospho-Akt (4060), Akt (4685), Phospho-p44/42 (9101) and p44/42 (9102), all purchased from Cell Signaling Technology (Beverly, MA, USA). After three washes in TBST, the blots were then incubated with anti-rabbit or anti-mouse IgG antibody conjugated to horseradish peroxidase for 1 h. The protein bands were visualised using an SuperSignal West Femto Maximum Sensitivity Substrate (Thermo Fisher Scientifc, Rockford, IL, USA). The membrane was then washed and reblotted with an actin antibody as an internal control. Densitometric quantification was performed using the ImageJ software (NIH, Bethesda, Maryland).

### Immunostaining and confocal imaging

Ten oocytes were fixed in 4% paraformaldehyde for 30 min and permeabilised for 30 min with PBS containing 0.1% Triton X-100. Permeabilised oocytes were blocked for 1 h at room temperature in 1% bovine serum albumin (BSA) and 0.1% Triton X-100 in PBS before overnight incubation at 4 °C with the primary antibodies for anti-α-tubulin (Cell Signaling Technology, Beverly, MA, USA), anti-HDAC6 and anti-acetyl-α-tubulin. The oocytes were washed several times in 0.05% Tween 20 in PBS (PBST), transferred to a secondary antibody mixture of Alexa Fluor 568 goat anti-mouse and Alexa Fluor 488 goat anti rabbit (Molecular Probes, USA), and incubated at room temperature for 30 min. Aggregates of ubiquitinated proteins (aggresomes) were detected in oocytes after treatment with Tub A using a ProteoStat Aggresome Detection Kit (Enzo Life Sciences, Inc., USA), and confocal images using the TO-PRO-3 fluorescent dye were acquired using an Olympus FV1000 Confocal microscope (Tokyo, Japan), and were processed using the FV10-ASW 2.0 Viewer software (Olympus, Tokyo, Japan). Fluorescent images were acquired using an Olympus BX-UCB microscope and were processed using a DP controller software (Olympus, Tokyo, Japan). The quantitative analysis of the fluorescent intensity was performed using the MeTaMorph image analysis software (Molecular Devices, California, USA).

### Relative mRNA quantification by Real-Time PCR

We collected 50 oocytes per group after 12 h culture. Total RNAs was extracted from oocytes using the Dynabeads mRNA Direct Kit (Thermo Fisher Scientifc, Rockford, IL, USA) according to the manufacturer’s instructions. Real-time PCR was conducted using a ViiA 7 Real-time PCR system (Applied Biosystems, OR, USA) and SYBR Green as the double-stranded DNA-specific fluorescent dye (Applied Biosystems, OR, USA). Target gene expression levels were normalised to *GAPDH* mRNA expression, which was unaffected by Tub-A treatment. The real-time PCR primer sets were: mTOR-F (5ʹ-CTC AGG CTG GAG CTT AT-3ʹ), mTOR-R (5ʹ-GCC AAA GCA CTG CAC TAC AA-3ʹ), mDia1-F (5ʹ-TCC AAG CTG ACA GGA GAG GT-3ʹ) and mDia1-R (5ʹ-GGG GGA GGT GGA ATA ACA GT-3ʹ) (Macrogen, Korea). Real-time PCR was performed independently in triplicate for each of the different samples, and the data are presented as the mean values of the gene expression levels measured in the Tub-A treated samples *versus* the controls.

### Fluorescence and western band intensity analysis

Fluorescence intensity was assessed using the Image J software (NIH, Bethesda, Maryland). For fluorescence intensity analysis, samples for control and treated oocytes were mounted on the same glass slide, and the same parameters were used to normalise across replicates. After immunofluorescent staining, the average fluorescence intensity per unit area within the region of interest (ROI) of immunofluorescence images was examined. Independent measurements using identically sized ROIs were taken from the cytoplasm. When calculating the fluorescence intensity, we ignored abnormal cells (little oocytes with extremely strong or weak signals). The average values for all measurements were used to determine the final average intensities for the control and the treated oocytes. To quantify the western blot results, the intensity values of the bands were measured using the Image J software (NIH, Bethesda, Maryland). Three different replicates were used for the analysis.

### Statistical analysis

At least three biological replicates were used for each analysis. Each replicate was prepared in an independent experiment, at a different time. Results are given as means ± SDs. Statistical comparisons were made using analysis of variance (ANOVA), and differences between treatments groups were assessed with Duncan’s multiple range test. A *P*-value of < 0.05 was considered significant.

## Electronic supplementary material

Supplementary Information
