# Supplementary material for: Histone deacetylase 6 (HDAC6) is an essential factor for oocyte maturation and asymmetric division in mice
Source: Sci Rep. 2017 Aug 15;7:8131. doi: 10.1038/s41598-017-08650-2 (PMC5557833; doi:10.1038/s41598-017-08650-2)
Supplement: Supplementary file 1 — Supplementary Information [file 41598_2017_8650_MOESM1_ESM.docx]

**Supplementary Data**

**Histone deacetylase 6 (HDAC6) is an essential factor for oocyte maturation and asymmetric division in mice**

Dongjie Zhou, Yun-Jung Choi, and Jin-Hoi Kim*

Department of Stem Cell and Regenerative Biotechnology, Humanized Pig Research Center (SRC), Konkuk University, Seoul 143-701, Republic of Korea

* Corresponding author: Department of Stem Cell and Regenerative Biology, Humanized Pig Research Center (SRC), Konkuk University, Seoul 143-701, Republic of Korea.

E-mail address: jhkim541@konkuk.ac.kr


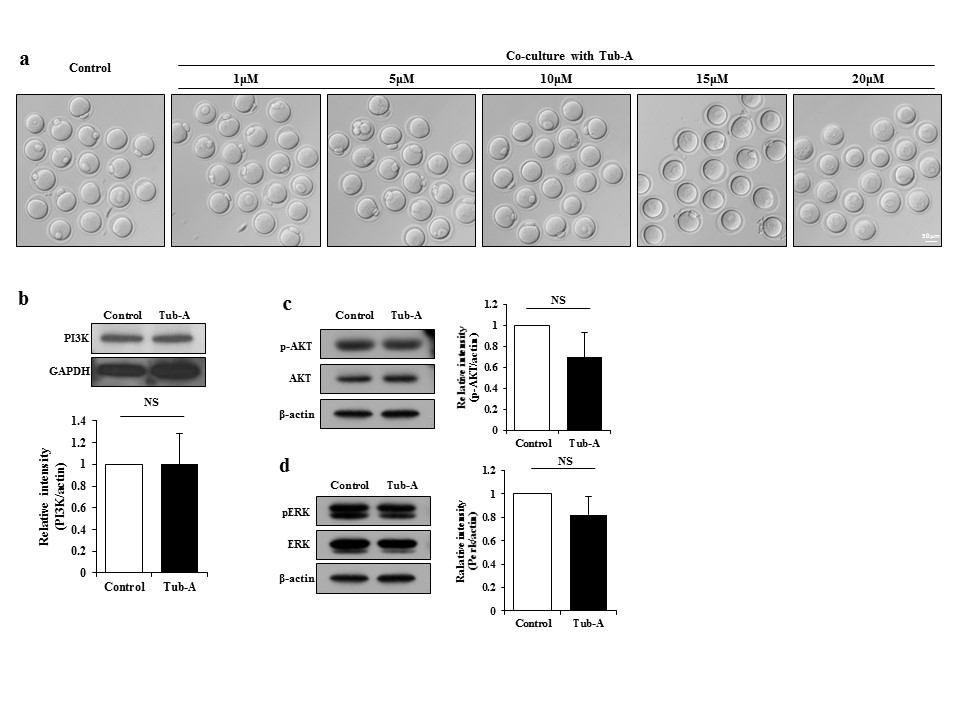


**Supplementary Figure 1.**

**a.** Images of control oocytes and of oocytes treated with Tub-A at different concentrations, at 12 h following the treatment. **b, c, d.** Protein expression of PI3 kinase, p-AKT and AKT, p-ERK and ERK, as detected by western blot. Band intensities were calculated using the ImageJ software; the ratio of each protein/actin expression was normalized, and the values are indicated. **p*<0.05, ***p*<0.01.


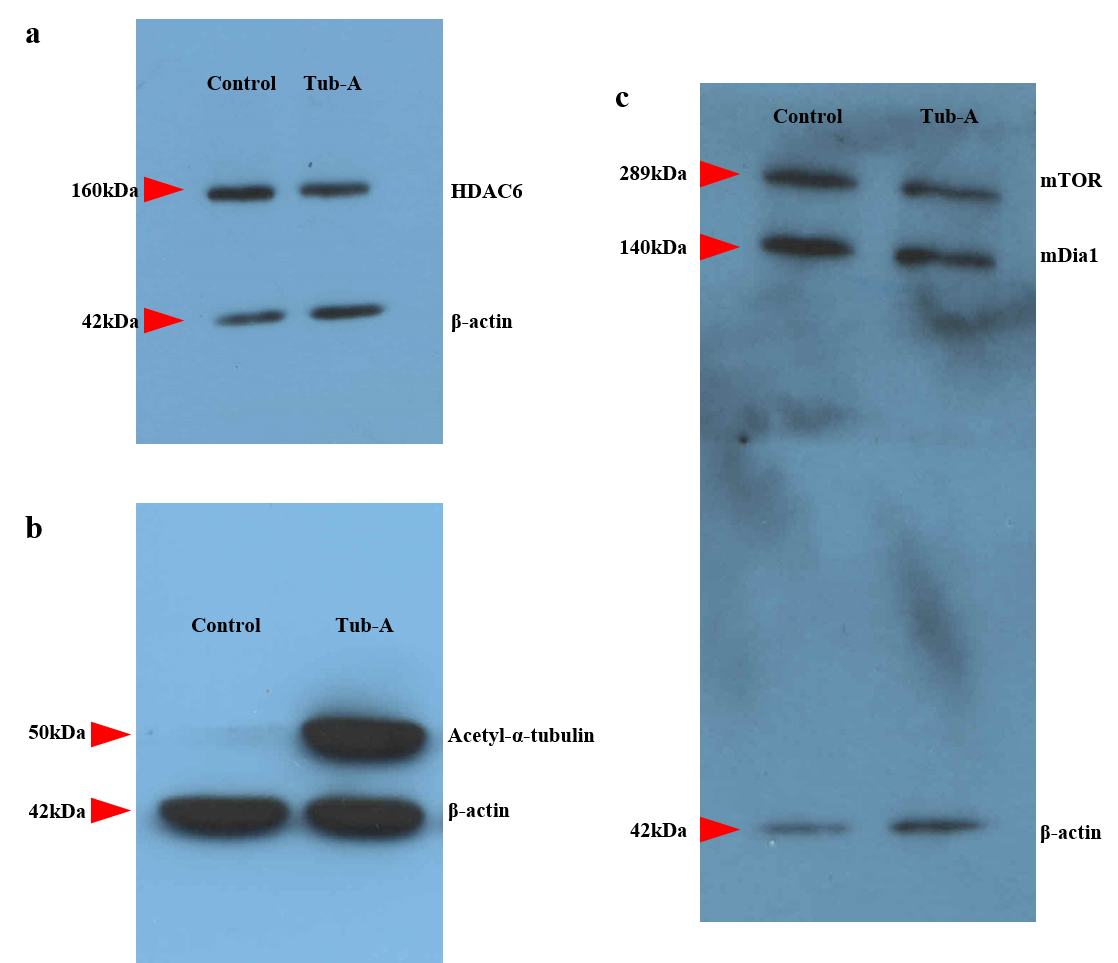


**Supplementary Figure 2. Full length gel images. a-c.** The figure is showing full length gel images of HDAC6, acetyl-α-tubulin, mTOR and mDia 1 western-blot data. The images are representative of three independent experiments.


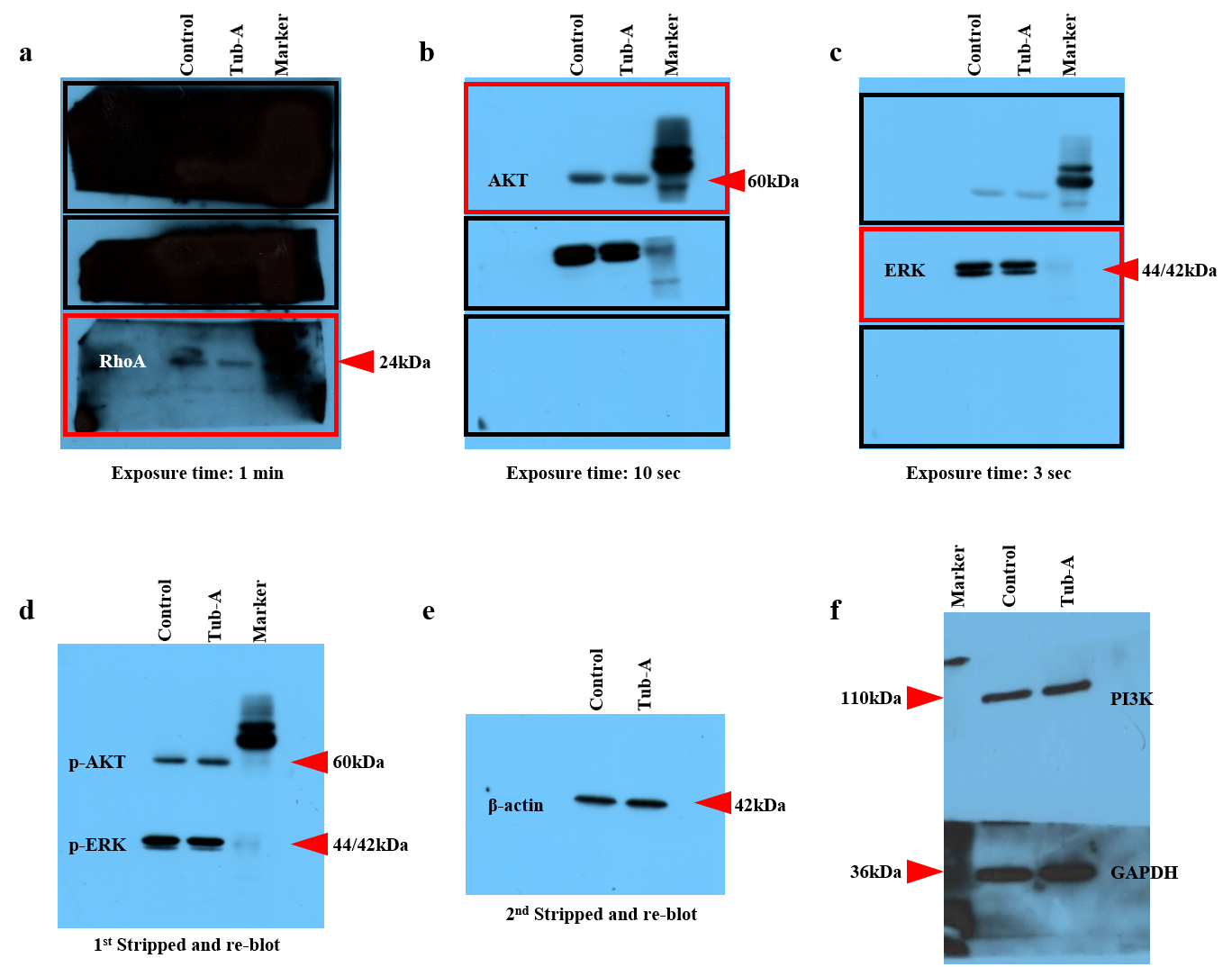


**Supplementary Figure 3. Full length gel images.**

**a-c.** The figures are showing full length gel images of RhoA, AKT and ERK western-blot data. Signal was detected with CL-XPosure Film exposed at the indicated times. **d.** Blots were stripped and re-bloted with phosphor-AKT and phosphor-ERK. Signal was detected with CL-XPosure Film exposed for 10 second. **e.** Blot was stripped again and re-blotted with β-actin. Signal was detected with CL-XPosure Film exposed for 5 second. **f.** The full length gel image of PI3K western-blot data. The images are representative of three independent experiments.
